# *N*^6^-methyladenosine (m^6^A)-circHECA facilitates the differentiation of SHF stem cells into hair follicle lineage through miR-449a-5p/LEF1 mediated Wnt/β-catenin pathway in cashmere goats

**DOI:** 10.5713/ab.250779

**Published:** 2026-05-07

**Authors:** Yixing Fan, Taiyu Hui, Qi Zhang, Ruqing Xu, Jincheng Shen, Yubo Zhu, Man Bai, Wenlin Bai

**Affiliations:** 1 College of Animal Science & Veterinary Medicine, Shenyang Agricultural University, Shenyang, China; 2 Engineering Research Center for Animal Molecular Genetics and Breeding of Liaoning Province, Shenyang, China

**Keywords:** Cashmere Goats, CircHECA, LEF1, N^6^-methyladenosine, MiR-449a-5p, SHF Stem Cells, Wnt, β-catenin Pathway

## Abstract

**Objective:**

This study investigates the potential effect of *N*^6^-methyladenosine (m^6^A)-circHECA on the differentiation of SHF stem cells into hair follicle lineage along with the underlying molecular mechanisms in cashmere goats.

**Methods:**

The effect of m^6^A-circHECA on the differentiation of SHF stem cells into hair follicle lineage was assessed through knockdown its expression in SHF stem cells derived from cashmere goats. The functional significance of m^6^A modification in circHECA functional exertion was confirmed through transfecting m^6^A deficient mutants into circHECA knockdown SHF stem cells. The competitive bindings of *miR-449a-5p* to m^6^A-circHECA and the 3′-untranslated region of *LEF1* mRNA was investigated using Dual-luciferase reporter assay.

**Results:**

The m^6^A-circHECA exhibited significantly higher expression in SHF stem cells post-differentiation than pre-differentiation. Moreover, m^6^A-circHECA facilitated the differentiation process of SHF stem cells into hair follicle lineages. The m^6^A-circHECA sequestering *miR-449a-5p*, to enhance the expression of the *LEF1* gene in SHF stem cells and activating the Wnt/β-catenin signaling pathway. We further demonstrated that the m^6^A modification within circHECA is necessary for the miR-449a-5p/LEF1 mediated Wnt/β-catenin pathway, which promote the differentiation of SHF stem cells into hair follicle lineages via the introduction of a m^6^A-deffcient mutant of circHECA.

**Conclusion:**

The m^6^A-circHECA facilitates the differentiation of SHF stem cells into hair follicle lineage through miR-449a-5p/LEF1 mediated Wnt/β-catenin pathway in cashmere goats.

## INTRODUCTION

Cashmere goats have been widely raised in agricultural and pastoral areas in northern China with great economic significance for local farmers and herdsmen [1]. As one of main products from cashmere goats, the cashmere has unique fiber qualities, such as smooth texture, lustrous color, and excellent warmth retention [2,3]. It is considered a high-grade raw material in textile industry, and its fabrics have highly been praised and favored by consumers [4]. Cashmere is produced from dynamic mini-organs called secondary hair follicles (SHFs) in the skin tissue of cashmere goats [5]. As well known, the periodic activity of cashmere goat SHFs undergoes three main stages, consisting of anagen, catagen, and telogen. During SHF anagen, the differentiation event of SHF stem cells into hair follicle lineages under the signal stimulation from dermal papilla cells (DPCs) is crucial for the regeneration of SHFs along with the morphogenesis and growth of cashmere in cashmere goats [6]. Previously, the regulatory mechanisms on the differentiation of hair follicle stem cells were extensively investigated at functional genes [7–9], and signaling pathway levels [10–12]. In fact, however, the differentiation of hair follicle stem cells into the hair follicle lineage is a complex physiological process where a variety of endogenous regulatory factors were implicated through a jointly coordinated regulatory network at multiple levels.

Over past few years, some non-coding RNAs were demonstrated to play significant roles in regulating the differentiation process of hair follicle stem cells into hair follicle lineages. It was reported that the *miR-22* was revealed to negatively regulate the differentiation process of hair follicle stem cells through inhibiting the *STK40* expression [13]. While the lncRNA *PlncRNA 1* was found to facilitate the differentiation of hair follicle stem cells into hair follicle lineages through TGF-β1-mediated Wnt/β-catenin signaling pathway [10]. Also, several circRNAs were recorded to promote the differentiation process of SHF stem cells through miRNA mediated axes in cashmere goats, such as circRNA-1926 via *miR-148a/b-3p/CDK19* axis [14], circRNA-0100 via *miR-153-3p/KLF5* axis [6], and circRNA-1967 via *miR-93-3p/LEF1* axis [15].

As well known, the *N*^6^-methyladenosine (m^6^A) is the most common post-transcriptional modification in eukaryotic linear RNAs including mRNAs, miRNAs, and lncRNAs [16,17]. Moreover, the m^6^A modification was essentially implicated in the functional exertion of the RNA molecules [18]. Interestingly, extensive m^6^A modification sites also were identified in many circRNAs molecules with functional significance [19,20]. It was demonstrated that m^6^A modification regulated the effect of circRNA-08436 on lipid metabolism of mammary glands in dairy goat [21]. The m^6^A modifications of circRNAs might also be largely implicated in the formation of pork quality [22], the infection of Marek’s disease virus in chicken [23], and the inflammation of mammary epithelial cells injured by *Staphylococcus aureus* and *Escherichia coli* in dairy cattle [24]. More recently, it was reported that the m^6^A modification of circCDK14 mediated by SRSF3 and hnRNP A1 plays a crucial role as an *miR-4492-z* sponge in regulating intramuscular fat deposition in yaks [25]. In cashmere goats, a considerable number of m^6^A modified circRNAs were identified from skin tissue or SHFs [5,26,27]. Moreover, the circRNA-ZNF638 and circERCC6 were proved to facilitate the activation process of SHF stem cells in cashmere goats through miRNA mediated pathways in m^6^A-dependent pattern [28,29].

The m^6^A modified circHECA (m^6^A-circHECA) was recently identified from cashmere goat SHFs with significantly higher expression at anagen SHFs compared with its counterpart of telogen, and its expression was detected mainly in cytoplasm of SHF stem cells of cashmere goats [30]. However, the potential functions of m^6^A-circHECA are unknown in SHF stem cells of cashmere goats. In this present study, we mainly investigated the effect of m^6^A-circHECA on the differentiation of SHF stem cells into hair follicle lineage in cashmere goats. Further, we explored the potential mechanisms of m^6^A-circHECA regulating the differentiation process of SHF stem cells. The results from this study would provide novel scientific evidence for unveiling the potential regulatory mechanisms on the differentiation of SHF stem cells into hair follicle lineage in cashmere goats.

## MATERIALS AND METHODS

### Cell co-culture and isolation of total RNA

Here, we utilized the SHF stem cells and DPCs of cashmere goats that have been stored in our laboratory [29]. The SHF stem cells were co-cultured (non-contact) with DPCs for inducing the differentiation to hair follicle lineages in a transwell devices as described in a previous publication [31]. In brief, the SHF stem cells were firstly seeded on six-well plates at a density of 1×10^4^ cells/mL, and then, a transwell insert was added in which we seeded the DPCs 1×10^4^ cells/mL. The two types cells (SHF stem cells and DPCs) were co-culture (non-contact) for 7 days in fresh DMEM/F12 medium (Hyclone). The incubator temperature was set as 37°C with the CO_2_ concentration of 5%. The medium was changed every 2 days. The total RNA from the harvested SHF stem cells was isolated using the RNAiso reagent kit following the manufacturer’s instructions (TaKaRa). In addition, for the expression analysis of circHECA and *miR-449a-5p* in SHFs of cashmere goats during different stages of SHF cycles, the total RNA was used that was extracted from SHFs of cashmere goats in our previous study [30].

### Analysis of knockdown and overexpression of circHECA in SHF stem cells

For the knockdown of circHECA in SHF stem cells, three siRNAs (si-circHECA-1^#^, si-circHECA-2^#^, and si-circHECA-3^#^) were designed based on the upstream and downstream sequence of back splicing site of circHECA. The three designed siRNAs to circHECA are si-circHECA-1^#^: 5′-GCACAAGG GGCTCTTGAAGGTG-3′, si-circHECA-2^#^: 5′-AA GGGGCTCTTGAAGGTGACAG-3′, and si-circHECA-3^#^: 5′-GTTGCACAAGGGGCTCTTGAAG-3′. These three siRNAs were transfected respectively into SHF stem cells using the Lipofectamine RNAiMAX kits (Invitrogen) according to the manufacturer’s instructions. For the overexpression of circHECA in SHF stem cells, the overexpression vectors of circHECA (or its mutants) were constructed using the pcDNA3.1 (+) circRNA mini-vector (Addgene). The circHECA mutants were generated by the QuikChange Lightning Multi Site-Directed Mutagenesis Kit (Agilent Technologies). The recombinant pcDNA3.1 (+) circHECA (or its mutant) was transfected into SHF stem cells by the Lipofectamine 3000 (Invitrogen) following the manufacturer’s instructions (Invitrogen).

### Dual-Luciferase reporter assays

In the present study, the dual-luciferase reporter experiments were performed according to the assays described by Yu et al. [32]. Briefly, to define the specific binding of circHECA with *miR-449a-5p*, we generated the luciferase reporters through ligating the circHECA (circHECA-WT) or its mutant (circHECA-MUT) into pGL3-basic vectors (Promega). The circHECA-MUT was generated by the QuikChange Lightning Multi Site-Directed Mutagenesis Kit (Agilent Technologies), where the potential binding sites of *miR-449a-5p* seed region were replaced with the corresponding complementary base. Similarly, to confirm the specific binding of the *LEF1* mRNA 3′-untranslated region (3′-UTR) with *miR-449a-5p*, the 3′-UTR fragment of cashmere goat *LEF1* mRNA (containing the putative binding region of miR-449a-5p) was amplified and further ligated into the pGL3 basic vector (Promega). The constructed reporter vectors were transfected into the 293T cells using the Lipofectamine 2000 (Invitrogen), respectively. The transfected 293T cells were cultured for 48 h. And then, the cells were collected, and lysed using lysate (Beyotime), followed by measurement of luciferase activity with the Dual-Luciferase Reporter Assay System (Promega). The relative ratio of firefly luciferase to Renilla luciferase activities was calculated to eliminate the potential bias from transfection efficiency among different samples.

### Overexpression and knockdown of *miR-449a-5p* and *LEF* mRNA

For the overexpression and knockdown of *miR-449a-5p*, its mimics and inhibitors were commercially obtained from Shanghai GenePharma with the corresponding negative controls (NC). For the overexpression of *LEF1* gene in SHF stem cells of cashmere goats, its overexpression vectors were generated where the cDNA was synthesized from the total RNA isolated from cashmere goat SHFs using PrimeScript 1st Strand cDNA Synthesis Kit (TaKaRa). For amplifying the coding sequence of *LEF1* mRNA, we designed a pair of primers according to the goat *LEF1* mRNA sequence (GenBank accession: XM_018049144.1). The polymerase chain reaction (PCR) amplifications were performed to obtain the coding sequence of goat *LEF1* mRNA by the Phanta Max Super-Fidelity DNA Polymerase (Vazyme). The amplified products were purified and cloned to the pcDNA3.1(+) vector. For the knockdown of *LEF* mRNA in SHF stem cells of cashmere goats, the small interference RNA targeting *LEF1* mRNA (siRNA-*LEF1*) were commercially obtained from Shanghai GenePharma with its negative control (siRNA-NC). The small RNA molecules were transfected into SHF stem cells using the Lipofectamine RNAiMAX kits according to the manufacturer’s instructions including mimics and inhibitors of *miR-449a-5p*, siRNA-*LEF1* and siRNA-NC (Invitrogen). The generated overexpression vector of *LEF1* mRNA was transfected into SHF stem cells of cashmere goats by the Lipofectamine 3000 following the manufacturer’s instructions (Invitrogen).

### Quantitative real-time polymerase chain reaction reactions

In qPCR analysis, all primers used are provided in Table 1. The qPCR reactions were carried out in a final volume of 25 μL consisting of Green Premix Ex Taq II of 12.5 μL TB (TliRNaseH Plus; TaKaRa), each primer of 1.0 μL (10 μM), the first-strand cDNA solution of 2.0 μL, and ddH_2_O water of 8.5 μL. The thermal cycling parameters were comprised of a single cycle of 95°C for 3 min, followed by 40 cycles of 9°C for 5 s, 53°C–60°C (Table 1) for 30 s, and 72°C for 30 s. The *GAPDH* was used as internal controls for expression analysis of circHECA and tested mRNAs, whereas the U6 was used as internal controls for expression analysis of *miR-449a-5p*. Ultimately, the relative expression of analyzed RNA molecules was calculated with the 2*^−ΔΔCt^* method [33].

### Data statistical analysis

The obtained data was presented as mean±standard error, and analyzed using the SPSS 17.0 procedure (SPSS). The graphical representations were performed using the GraphPad Prism 8 software (GraphPad Software). The mean difference between analyzed groups was compared by the unpaired Student’s t-test. The obtained p-values<0.05 were considered significant statistically.

## RESULTS AND DISCUSSION

### Expression characterization of m^6^A-circHECA in SHF stem cells before and after differentiation, and its effects on the differentiation into hair follicle lineages

**Figure 1:**
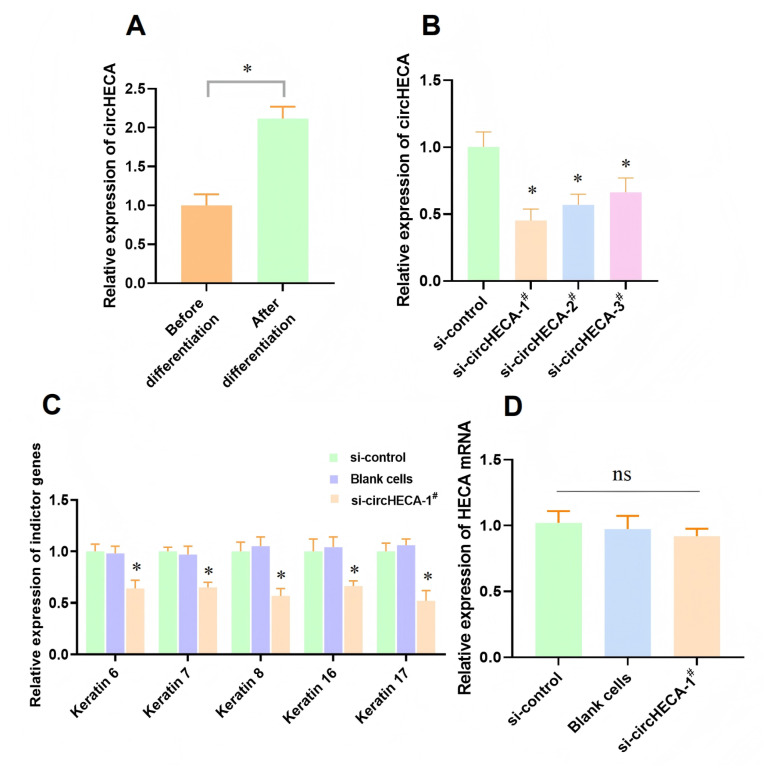
The relative expression of m^6^A-circHECA in SHF stem cells before and after differentiation with its functional significance in the differentiation of SHF stem cells into hair follicle lineages in cashmere goats. (A) The relative expression of circHECA in SHF stem cells before and after differentiation into hair follicle lineages. (B) Knockdown efficiency analysis of si-circHECA-1^#^, si-circHECA-2^#^ and si-circHECA-3^#^ to circHECA in SHF stem cells of cashmere goats. (C) Knockdown of circHECA led to the significant decrease in expression level of the analyzed indicator genes in SHF stem cells. (D) Effect of circHECA on the expression of its host HECA gene. ‘*’ represents the significant difference compared with the si-control, p<0.05. ‘ns’ represents no significant difference, p>0.05. The ‘blank cells’ refers to the group of cells that did not receive any treatment, and it serves as a baseline to indicate the expression level of related molecules in native, unmodified cells. The ‘si-control’ was introduced as a control for ‘si-circHECA-1^#^’.

To explore the potential role of m^6^A-circHECA in the differentiation of SHF stem cells into hair follicle lineages, firstly, we investigated its expression changes in SHF stem cells before and after differentiation. As shown in Figure 1A, m^6^A-circHECA exhibited significantly higher expression in SHF stem cells after differentiation than that before differentiation. Thus, it is reasonable to assume that m^6^A-circHECA may play certain role in the differentiation process of SHF stem cells into hair follicle lineages in cashmere goats. To define the hypothesis, we carried out a knockdown analysis of m^6^A-circHECA in SHF-stem cells through siRNA interference experiments. We designed three independent siRNAs and named as si-circHECA-1^#^, si-circHECA-2^#^ and si-circHECA-3^#^. Based on their knockdown efficiency to m^6^A-circHECA in SHF-stem cells of cashmere goats (Figure 1B), the si-circHECA-1^#^ was chosen and utilized in further knockdown experiment of m^6^A-circHECA. As shown from Figure 1C, the si-circHECA-1^# -^mediated knockdown of m^6^A-circHECA led to a significant decrease in the expression of several indicator genes on upon SHF-stem cell differentiation (including *Keratin 6*, *Keratin 7*, *Keratin 8*, *Keratin 16* and *Keratin 17*) in comparison to their counterparts of the blank cells and negative control group (si-control) (Figure 1C). These results suggest that m^6^A-circHECA may facilitate the differentiation process of SHF stem cells into hair follicle lineages via certain mechanisms. Here, the Keratins 6,7,8,16,17 were chosen as biomarkers of SHF differentiation into hair follicle lineages based on the previous publications where the biomarker role of Keratin 6,7,8,16,17 in SHF differentiation into hair follicle lineages was described in detail [6,14,15].

However, the knockout of m^6^A-circHECA had no significantly affect on the expression of its host gene *HECA* in SHF stem cells (Figure 1D), indicating that the host gene HECA of m^6^A-circHECA appears not to be involved in the observed effect of m^6^A-circHECA on the differentiation process of SHF stem cells into hair follicle lineages. To date, it is still unclear how m^6^A-circRNAs regulate the cell-fate decision of SHF-stem cells of cashmere goats at epigenetic level. Interestingly, there is growing evidence that m^6^A modification is closely related to the functional exertion of m^6^A-circRNAs [34,35]. As described in our recent study, four m^6^A modified sites were verified within the circHECA sequence, including m^6^A-213, m^6^A-297, m^6^A-780 and m^6^A-927 [30]. This drives us to ask whether the observed effect of m^6^A-circHECA on facilitating the differentiation process of SHF stem cells into hair follicle lineages was achieved through relying on its m^6^A modifications.

### The m^6^A modification of circHECA is necessary in it facilitating the differentiation of SHF stem cells into hair follicle lineages

**Figure 2:**
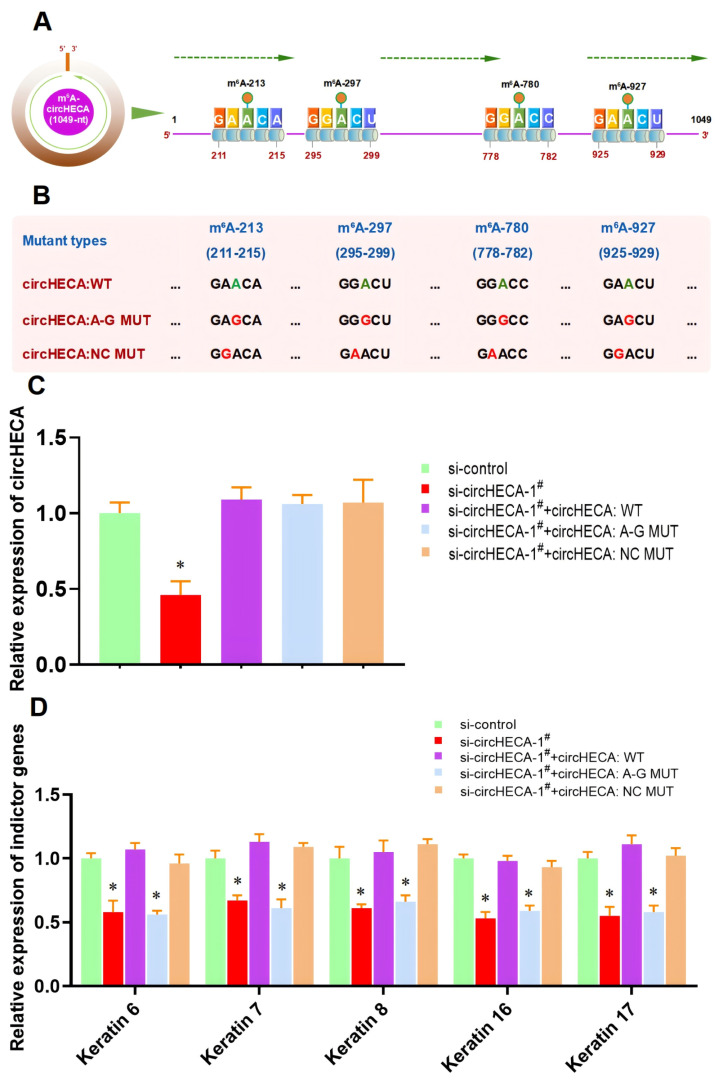
m^6^A-circHECA contributes the differentiation of SHF stem cells into hair follicle lineages in cashmere goats through relying on m^6^A modifications. (A) Overall diagram of m^6^A sites within circHECA of cashmere goat SHFs including m^6^A-213, m^6^A-297, m^6^A-780, and m^6^A-927. (B) The generation strategies for circHECA mutants against its m^6^A sites. circHECA:WT = wild type of circHECA, circHECA:A-G MUT = circHEAC mutant against the m6A sites, and circHECA:NC MUT = circHECA negative control mutant against the m^6^A sites. (C) The effects of m^6^A sites mutation on circHECA expression in SHF stem cells with circHECA knockdown. (D) The effects of m^6^A site mutation of circHECA on the expression of indicator genes in SHF stem cells with circHECA knockdown. The ‘*’ represents significant difference compared with si-control, p<0.05. The ‘si-control’ was introduced as a control for ‘si-circHECA-1^#^’. The ‘circHECA: NC MUT’ was introduced as a control for ‘circHECA: A-G MUT’.

To confirm the above hypothesis, through pointing at the four verified m^6^A sites (m^6^A-213, m^6^A-297, m^6^A-780 and m^6^A-927) of (Figure 2A) of m^6^A-circHECA, we constructed the wild type vectors of m^6^A-circHECA (circHECA: WT) and its mutation vectors (circHECA: A-G MUT) where the A-213, A-297, A-780 and A-927 in the m^6^A motif: RRACH were replaced with G-213, G-297, G-780 and G-927 respectively (Figure 2B). Also, a negative control mutant vectors (circHECA: NC MUT) was constructed in which the A-212, G-296, G-779 and A-926 within the m^6^A motifs: RRACH were replaced with G-212, A-296, A-779 and G-926, respectively (Figure 2B).

In order to assess the potential role of m^6^A modification in functional exertion of m^6^A-circHECA in SHF stem cells, we transfected the circHECA: WT, circHECA: A-G MUT, or circHECA: NC MUT into circHECA-knockdown SHF stem cells with equal amounts, and further utilized them in the induced differentiation assays *in vitro*. As a result, the introduction of circHECA mutations did not lead to a significant change in its expression level in SHF stem cells (Figure 2C). Also, we noted that the decreasing expression of circHECA in circHECA-knockdown SHF stem cells was restored through the introduction of circHECA: WT or its mutants (circHECA: A-G MUT, or circHECA: NC MUT) (Figure 2C). Further, we evaluated the expression of the indicator genes (upon the differentiation of SHF stem cells) in the differently transfected cell lines. We found that the ‘si-circHECA-1^#^+circHECA: WT’ cell lines, not the ‘si-circHECA-1^#^+circHECA: A-G MUT’ cell lines, were significantly higher in expression abundance of the detected indicator genes, in comparison to the ‘si-circHECA-1^#^ cell lines (Figure 2D). Here, we can rule out that the observed expression changes of the analyzed indicator genes between the two types of cell lines (‘si-circHECA-1^#^+circHECA: WT’ and ‘si-circHECA-1^#^+circHECA: A-G MUT’) are from the expression abundance of circHECA in the tested cell lines, as there is no significant difference in expression abundance of circHECA between the two types of cell lines (‘si-circHECA-1^#^+circHECA: WT’ and ‘si-circHECA-1^#^+circHECA: A-G MUT’) (Figure 2C). In addition, it is worth noting that the circHECA: NC MUT (m^6^A-decorated mutant) restored the decreasing expression of the indicator genes in circHECA m^6^A-deficient cell lines (Figure 2D). Taking into account these above results, we can draw a conclusion that the m^6^A modification of circHECA is necessary in its facilitating the differentiation of SHF stem cells into hair follicle lineages. Such significant roles of internal m^6^A modification within non-coding RNAs in their function exertion were also reported in linc1281 of mouse embryonic stem cells [36], as well as, circRNA-ZFN638 [28] and circERCC6 [29] of goat SHF stem cells.

### The m^6^A-circHECA serves as *miR-449a-5p* sponge and may regulate its expression in SHF stem cells of cashmere goats

**Figure 3:**
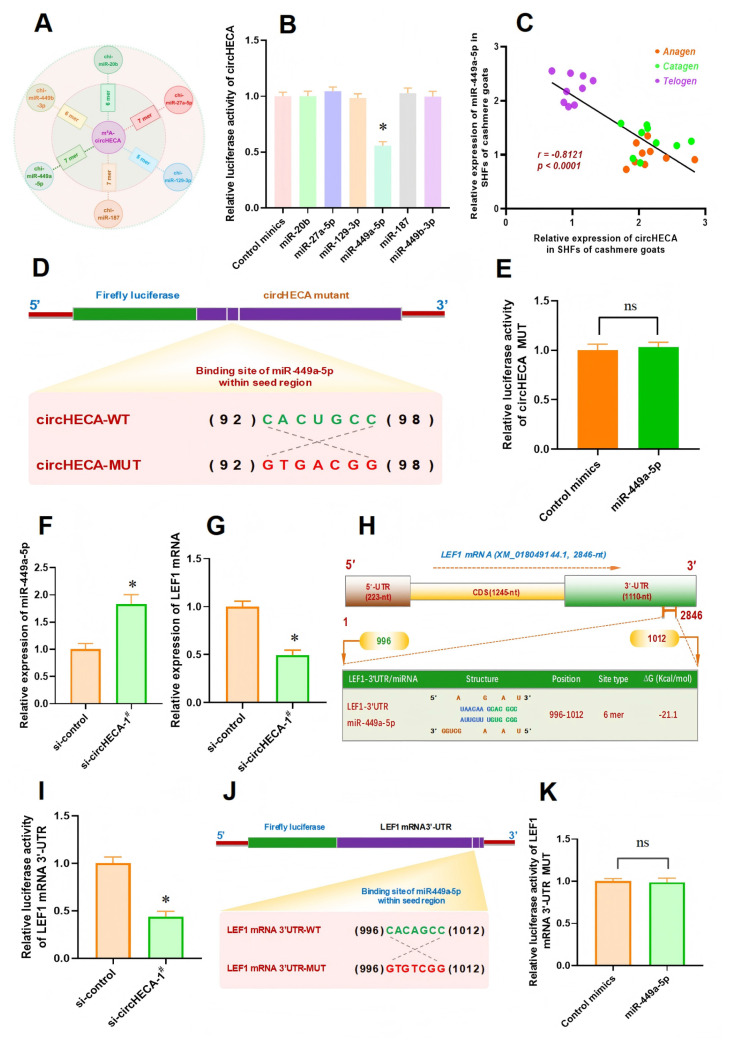
The m^6^A-circHECA sequesters *miR-449a-5p* to enhance the *LEF1* expression SHF stem cells. (A) Based on *in silico* analysis, the potential target miRNAs binding with m^6^A-circHECA including *miR-20b*, *miR-27a-5p*, *miR-129-3p*, *miR-449a-5p*, *miR-187*, and *miR-449b-3p*. (B) Relative luciferase activities of reporters including circHECA in 293T cells after 48 h co-transfection with the predicated different miRNA mimics, respectively. (C) Expression correlation analysis of m^6^A-circHECA and *miR-449a-5p* in SHFs of cashmere goats at anagen, telogen, and catagen. (D) The generating manners of circHECA mutant (circHECA-MUT) for the binding sites of *miR-449a-5p* within circHECA. (E) Relative luciferase activities of reporters containing circHECA mutant (circHECA MUT) in 293T cells after 48 h co-transfection with the control mimics or *miR-449a-5p* mimics. (F) Relative expression of *miR-449a-5p* in SHF stem cells with circHECA knockdown. (G) Relative expression of *LEF1* mRNA in SHF stem cells with circHECA knockdown. (H) A diagram of goat *LEF1* mRNA with the *in silico* analysis on binding sites of *miR-449a-5p* within the 3′-UTR of *LEF1* mRNA. The nucleotide positions were indicated following the goat *LEF1* mRNA sequence at NCBI (https://www.ncbi.nlm.nih.gov) with accession number: XM_018049144.1. (I) Relative luciferase activities of reporters containing *LEF1* mRNA 3’-UTR in SHF stem cells with circHECA knockdown. (J) The generating manners for 3’-UTR mutant of *LEF1* mRNA (*LEF1* mRNA3’-UTR-MUT) for the *miR-449a-5p* binding sites. (K) Relative luciferase activities of reporters including *LEF1* mRNA-3’UTR mutant (LEF1 mRNA 3’UTR-MUT) in 293T cells after 48 h co-transfection with the control mimics or *miR-449a-5p* minics. The ‘*’ represents significant difference compared with the si-control, p<0.05. The ‘ns’ represents no significant difference, p>0.05.

It is widely believed that most circular RNAs located in the cytoplasm can sequester natural miRNAs to further regulate the expression of corresponding target genes [37]. In previous study, it was verified that m^6^A-circHECA was mainly expressed in cytoplasm of SHF stem cells in cashmere goats [30]. As shown in Figure 3A, moreover, m^6^A-circHECA had potential binding relationships with several miRNAs, including *chi-miR-449a-5p*, *chi-miR-129-3p*, *chi-miR-187*, *chi-miR-449b-3p*, *chi-miR-20b*, and *chi-miR-27a-5p* [30]. To define which miRNAs can directly bind to m^6^A-circHECA, a luciferase reporter was generated which contained the linear full-length sequence of m^6^A-circHECA in the downstream of the firefly luciferase gene. We co-transfected the generated reporter into 293T cells with the predicted target miRNA mimics, respectively. As a result, we found that only the *miR-449a-5p* co-transfected cell lines exhibited the significantly decreasing luciferase activities of m^6^A-circHECA reporters, but not for the other miRNAs (*chi-miR-129-3p*, *chi-miR-187*, *chi-miR-449b-3p*, *chi-miR-20b*, and *chi-miR-27a-5p*) co-transfected cell lines (Figure 3B). Moreover, in SHFs of cashmere goats, we found that a significantly negative correlation relationship existed in expression pattern between m6A-circHECA and *miR-449a-5p* during SHF cycles: telogen, anagen and catagen (Figure 3C).

To further validate this binding of m^6^A-circHECA and *miR449a-5p*, we constructed m^6^A-circHECA mutation luciferase reporters (circHECA MUT) that harbored an anti-sense mismatch of 7-nt long at the seed binding region of *miR-449a-5p* (Figure 3D). After co-transfection into 293T cells with *miR-449a-5p*, we found that the introduction of circHECA MUT reporters restored the *miR-449a-5p*-driven suppression in luciferase activities of m^6^A-circHECA reporters (Figure 3E). Taken together, these results provided evidence that m^6^A-circHECA might serve as *miR-449a-5p* sponge in the SHF stem cells of cashmere goats. On the other hand, we found that the knockdown cell lines of m^6^A-circHECA exhibited a significant increasing expression of *miR-449a-5p* in SHF-stem cells in comparison to the negative control cell lines (si-control) (Figure 3F). On the contrary, the knockdown of *miR-449a-5p* had no significant effect on the expression of m^6^A-circHECA in SHF stem cells of cashmere goats (data not shown). Thus, it can be suggested that m^6^A-circHECA may negatively regulate the *miR-449a-5p* expression in SHF-stem cells. Such regulatory mode was also reported in osteosarcoma cells where circ_0081001 bound with *miR-494-3p* and negatively regulated its expression [38]. Also, an investigation on glioma, Ke et al. [39] found that circ_0076931 can bind with *miR-6760-3p*, and negatively regulated its expression glioma cells.

### The m^6^A-circHECA upregulates the expression *LEF1* gene in SHF stem cells via *miR-449a-5p* mediated mechanism

As is well known, the canonical Wnt signaling pathway plays a crucial role in the morphogenesis, development and growth of hair follicles [40]. Whereas, *LEF1* is a key transcription factor that is deeply implicated in the activity of canonical Wnt signaling pathway. In previous investigations, it has been shown that *LEF1* can facilitate the differentiation of SHF stem cells into hair follicle lineages under the activated status of Wnt signaling pathway [15,41]. This drove us to ask whether the *LEF1* might be involved in the revealed effect of m^6^A-circHECA on the differentiation of SHF stem cells into hair follicle lineages (Figure 1C). Thus, we further analyzed the *LEF1* expression in m^6^A-circHECA knockdown SHF stem cells. As a result, we found that the si-circHECA-1^#^ mediated knockdown of m^6^A-circHECA led to a significant decreasing of *LEF1* expression in the analyzed cells (Figure 3G). Interestingly, we have verified that m^6^A-circHECA can sponge *miR-449a-5p* (Figures 3B, 3D, 3E). Moreover, the *LEF1* was also predicted as a potential target gene of *miR-449a-5p* in cashmere goats [30]. These findings raised a most likely mechanism that m^6^A-circHECA might positively regulate the *LEF1* expression in SHF stem cells via *miR-449a-5p*-mediated pathway.

To ascertain this hypothesis, firstly, we screened the potential binding sites of *miR-449a-5p* within the 3’-UTR region of *LEF1* mRNA based on *in silico* analysis. As a result, a potential binding site of *miR-449a-5p* was revealed in 3’-UTR region of goat *LEF1* mRNA with a 6 mer binding type of seed region and −21.1 ΔG (Kcal/mol) (Figure 3H). Secondly, we generated a luciferase reporter of 3’-UTR region of *LEF1* mRNA containing the potential binding site for *miR-449a-5p*, and co-transfected into the SHF stem cells with si-circHECA-1^#^, respectively. We noted that the knockdown of m^6^A-circHECA (si-circHECA-1^#^) significantly decreased the relative luciferase activity of *LEF1* mRNA 3’-UTR in SHF stem cells in comparison to siRNA control (si-control) (Figure 3I). Further, we generated a mutant luciferase report (*LEF1* mRNA 3’-UTR-MUT) that contained an antisense mismatch region of 7-nt long against seed region of *miR-449a-5p* (Figure 3J), co-transfected into SHF-stem cells with *miR-449a-5p* mimics. As shown in Figure 3K, there is no significant difference in relative luciferase activity of mutation reporters between *miR-449a-5p* mimics and the control mimic cell lines. These results indicated that the *miR-449a-5p* directly bound with *LEF1* mRNA 3’-UTR in SHF stem cells of cashmere goats.

Taking into account these above results, it appears to become apparent that m^6^A-circHECA upregulates the *LEF1* expression through sequestering *miR-449a-5p* in SHF stem cells of cashmere goats. This is a canonical regulatory mode for the function exertion of many circRNA molecules [37]. In fact, however, circRNAs can play biological roles in cells via multiple regulatory mode, such as regulating the transcription of corresponding host gene [42], RNA alternative splicing and maturation, protein scaffolding and localization [43], and acting as templates for protein translation [44]. Therefore, we strongly recommended that the other regulatory pathways of m^6^A-circHECA should be further investigated in SHF stem cells, which may have further implications for understanding the biological functions of m^6^A-circHECA in SHF stem cells of cashmere goats.

### The m^6^A modification is necessary for m^6^A-circHECA biological function through miR-449a-5p/LEF1 pathway

Increasing lines of evidence have demonstrated that m6A modifications in RNA molecules play critical roles in RNA splicing, maturity, transportation, stability and translation [45,46]. Although, the biological roles of m^6^A modification in circRNAs still needs to be elucidated, there is evidence that m^6^A modification in some non-coding RNAs were necessary in their interactions with target miRNAs, such as linc1281 with let-7 family [36], circRNA-ZNF638 and *miR-361-5p* [28], and circERCC6 and *miR-412-3p* [29]. Thus, we have reason to ask whether m^6^A modification of circHECA is necessary for its the observed effect on the differentiation of SHF stem cells through miR-449a-5p/LEF1 pathway.

**Figure 4:**
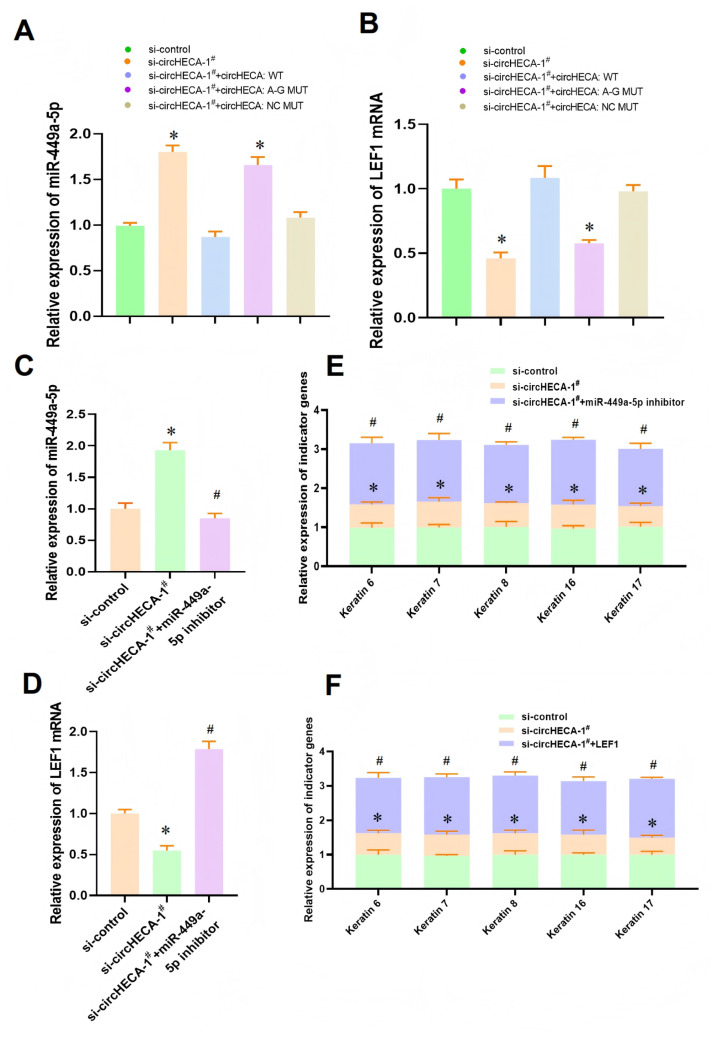
The m^6^A modification within circHECA is necessary for its functional role through miR-449a-5p/LEF1 axis that restored the differentiation of SHF stem cells into hair follicle lineages with circHECA-deficiency. (A, B) The effects of m^6^A sites mutations of circHECA on the expression of *miR-449a-5p* and *LEF* mRNA in SHF stem cells with circHECA knockdown, respectively. (C, D) The effects of *miR-449a-5p* inhibitor on the expression of *miR-449a-5p* and *LEF1* mRNA in SHF stem cells with circHECA knockdown. (E, F) Both *miR-449a-5p* inhibitor and *LEF* overexpression led to significant increased expression of the indicator genes in SHF stem cells with circHECA knockdown. ‘*’ represents significant difference compared with the si-control, p<0.05, whereas ‘#’ represents significant difference compared with the si-circHECA-1^#^, p<0.05. The ‘si-control’ was introduced as a control for ‘si-circHECA-1^#^’. The ‘circHECA: NC MUT’ was introduced as a control for ‘circHECA: A-G MUT’.

To validate this hypothesis, firstly, we investigated expression changes of *miR-449a-5p* in ‘si-circHECA-1^#^+ circHECA:WT’, ‘si-circHECA-1^#^+circHECA: A-G MUT’, and ‘si-circHECA-1^#^+circHECA: NC MUT’ transfected SHF stem cell lines, respectively. We found that both ‘si-circHECA-1^#^+circHECA:WT’ and ‘si-circHECA-1^#^+circHECA: NC MUT’ led to significant decreasing in *miR-449a-5p* expression compared with ‘si-circHECA-1^#^’ cell lines (Figure 4A). On the contrary, the ‘si-circHECA-1^#^+circHECA: A-G MUT’ cell lines still showed high levels of miR-449a-5p expression (Figure 4A), while it should be noted that circHECA transcript abundances had no significant difference among the different treated cell lines (Figure 2C). Secondly, we investigated the expression changes of *LEF* mRNA in the different treated cell lines. As shown in Figure 4B, both ‘si-circHECA-1^#^+circHECA:WT’ and ‘si-circHECA-1^#^+circHECA: NC MUT’ led to significant increasing in LEF1 mRNA expression compared with ‘si-circHECA-1^#^’ cell lines (Figure 4B), whereas the ‘si-circHECA-1^#^+circHECA: A-G MUT’ cell lines still showed low levels of *LEF1* mRNA expression (Figure 4B). Based on above these results, it can be suggested that the m^6^A modification of circHECA is necessary for its the observed effect on the differentiation of SHF stem cells through miR-449a-5p/LEF1 pathway.

Currently, it is generally believed that the specific binding of miRNAs to circRNA/lncRNAs is driven by the base pairing based on nucleic acid sequences, however, it has remained elusive that whether there is any mediator to regulate this binding process [29,36]. Interestingly, the presence of m^6^A peak at the binding region of mRNAs with miRNAs suggests a most likely mechanism that m^6^A modification within mRNAs may collaborate with the corresponding miRNAs to ultimately regulate the mRNA expression in biological cells [47]. In the present work, we showed that the the m^6^A modification of circHECA was responsible for its binding with *miR-449a-5p*, which has been verified by the m^6^A-deficient A-G mutant of circHECA that hindered the binding of circHECA with *miR-449a-5p* (Figures 4A, 4B). Thus, although it still needs further clarification whether m^6^A modification of circHECA alters its local structure as previously described by Liu and colleagues [48] thereby facilitating the binding of circHECA with *miR-449a-5p*, our results provided valuable insights into the regulatory model of circRNA relying on m^6^A modification to promote the differentiation of SHF stem cells into hair follicle lineages in cashmere goats.

### The miR-449a-5p/LEF1 axis restores the differentiation of SHF stem cells into hair follicle lineages with m^6^A-circHECA deficiency

Given the above results, we further investigative whether the miR-449a-5p/LEF1 axis is responsible for the positive effect of m^6^A-circHECA on the differentiation of SHF stem cells into hair follicle lineages through inhibiting *miR-449a-5p* abundance in SHF stem cells with m^6^A-circHECA knockdown. As shown in Figure 4C, the introduction of *miR-449a-5p* inhibitor resulted in a significant decrease in *miR-449a-5p* abundance in the m^6^A-circHECA knockdown cells, whereas the *LEF1* mRNA abundance was significantly upregulated by the *miR-449a-5p* inhibitor in transfected cells (Figure 4D). On the other hand, we noted that the inhibition of *miR-449a-5p* could restore the restricted differentiation of SHF stem cells into hair follicle lineages after m^6^A-circHECA knockdown, which could be determined via the significant increase in expression level of the indicator genes (Figure 4E).

In addition, given *LEF1* mRNA was subjected to the negative regulation by *miR-449a-5p* (Figures 3H, 3K), moreover, the its expression was significantly down-regulated in SHF stem cells with m^6^A-circHECA knockdown (Figure 4B). Therefore, we want to know if *LEF1* promotes the potential mechanism of m^6^A-circHECA/miR-449a-5p regulatory model. For this purpose, we overexpressed the *LEF1* in SHF stem cells with m^6^A-circHECA knockdown. As a result, the overexpression of *LEF1* restored the restricted differentiation of SHF stem cells into hair follicle lineages resulting from m^6^A-circHECA deficiency, which can be determined by the significant upregulation in expression abundance of the indicator genes in transfected SHF-stem cells (Figure 4F).

In previous studies, it was demonstrated that *LEF1* expression was significantly upregulated at anagen phase during the hair follicle cycle [49], and *LEF1* could guide the special organization of hair follicles with the regulation on the fate of epithelial cells [50]. Also, the *LEF* binding with TCF protein regulates many genes implicated in hair follicle development [51], and *LEF*1 promotes the SHF maturation [52]. The overexpression of *LEF1* facilitates the proliferation of hair follicle stem cells and inhibits their apoptosis, whereas knockout of *LEF1* reverses these effects [53]. These studies demonstrated the impact of *LEF1* on the growth and development of hair follicles. Here, we showed that the *LEF1* is eventually responsible for the m^6^A-circHECA role in contributing the differentiation of SHF stem cells into hair follicle lineages via the *miR-449a-5p* mediated model, in which the m^6^A modification of circHECA is necessary. Our this result further supported the previous findings that *LEF1* contributed the differentiation of bulge stem cells toward a hair fate [54].

### CircHECA relying on m^6^A modification to activate the Wnt/β-catenin pathway through miR-449a-5p/LEF1 axis

**Figure 5:**
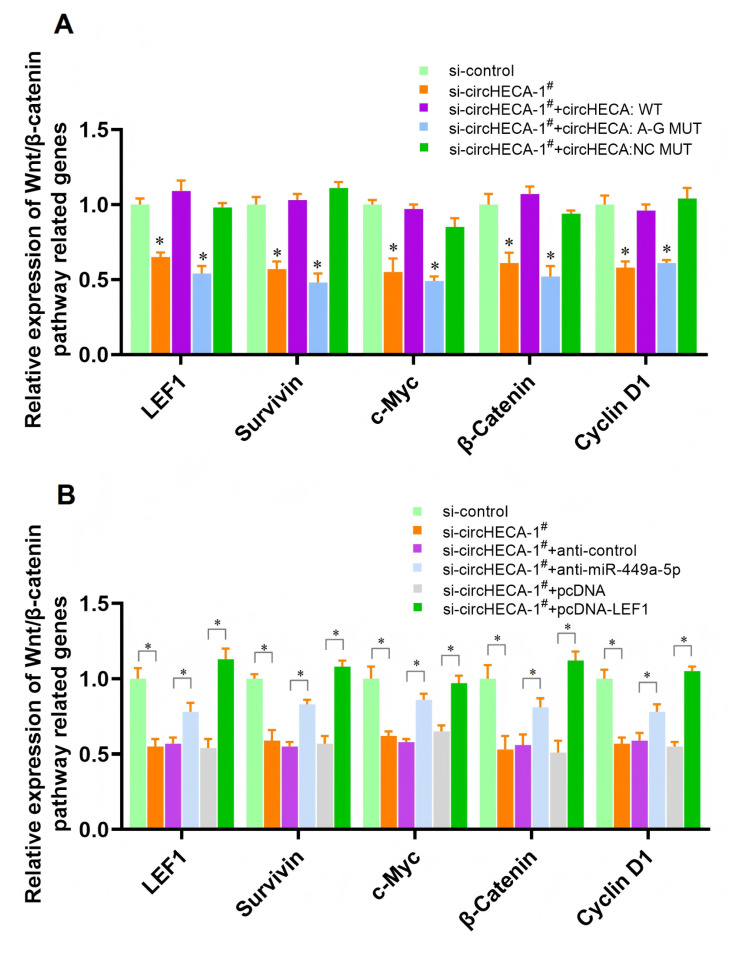
CircHECA relying on m^6^A modification to activate the Wnt/β-catenin pathway through miR-449a-5p/LEF1 axis. (A) The effects of m^6^A site mutation within circHECA on the expression of Wnt/β-catenin pathway related genes in SHF stem cells with circHECA knockdown. (B) The m^6^A-circHECA activates Wnt/β-catenin pathway through miR-449a-5p/LEF1 axis. The ‘*’ represents significant difference between the compared groups, p<0.05. The ‘si-control’ was introduced as a control for ‘si-circHECA-1^#^. The ‘anti-control’ was introduced as a control for ‘anti-miR-449a-5p’. The ‘circHECA: NC MUT’ was introduced as a control for ‘circHECA: A-G MUT’. The ‘pcDNA’ was introduced as a control for ‘pcDNA-LEF1’.

As is well known, the *LEF1* is a vital transcriptional factor of the Wnt/β-catenin signaling pathway that guides the fate of hair follicle stem cells in which the *LEF1* plays a key role via facilitating the nuclear translocation of *β-catenin* [50,54]. Given the association of *LEF1* with m^6^A-circHECA function in differentiation of SHF stem cells in cashmere goats, we further investigated the effect of circHECA relying on m^6^A modification on the Wnt/β-catenin pathway through miR-449a-5p/LEF1 axis, which was evaluated by testing several keys signaling molecules of Wnt/β-catenin pathway including *LEF1*, *Survivin*, *c-Myc*, *β-catenin*, and *Cyclin D1*. As shown in Figure 5A, the ‘si-circHECA-1^#^+circHECA: WT’ cell lines, not the ‘si-circHECA-1^#^+circHECA: A-G MUT’ cell lines, were significantly higher in mRNA expression of the tested genes, in comparison to the ‘si-circHECA-1^#^’ cell lines, while the circHECA: NC MUT (m^6^A-decorated mutant) restored the decreasing expression of the tested genes in circHECA m^6^A-deficient cell lines (Figure 5A). These results showed that circHECA activated the Wnt/β-catenin pathway where the m^6^A modification within circHECA molecule was required.

Further, we explore whether the effect of m^6^A-circHECA on activating Wnt/β-catenin pathway may be achieved through miR-449a-5p/LEF1 axis. We transfected the *miR-449a-5p* inhibitor or pcDNA-LEF1 in SHF stem cells with m^6^A-circHECA knockdown. As shown in Figure 5B, the knockdown of m^6^A-circHECA significantly downregulated the mRNA expressions of Wnt/β-catenin pathway related genes (*LEF1*, *Survivin*, *c-Myc*, *β-catenin*, and *Cyclin D1*) in comparison to the corresponding control cell lines (si-circHECA-1^#^+anti-control). Interestingly, these effects were counterbalanced through the introduction of *miR-449a-5p* inhibitor or pcDNA-LEF1 (Figure 5B). Taken together above these results, it can be inferred that circHECA relying on m^6^A modification to activate the Wnt/β-catenin pathway through sponging *miR-449a-5p* mediated *LEF1* upregulation.

**Figure 6:**
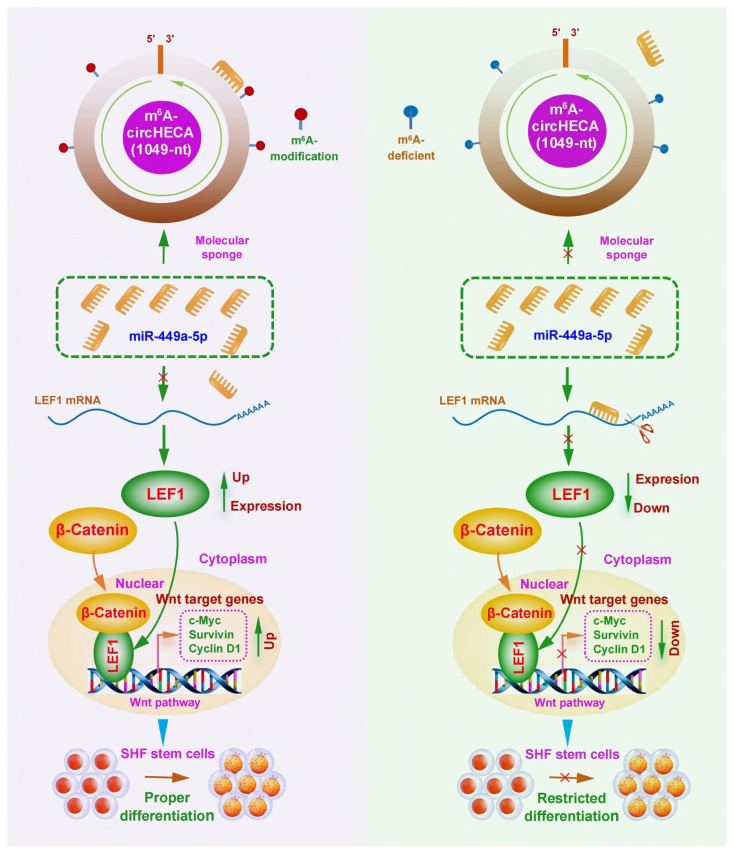
A schematic representation of circHECA relying on m^6^A modification to facilitate the differentiation of SHF stem cells into hair follicle lineages through miR-449a-5p/LEF1 mediated Wnt/β-catenin pathway in cashmere goats.

Over the past few years, increasing regulatory factors have been discovered for the differentiation of SHF stem cells into hair follicle lineages in cashmere goats [6,14,15]. Herein, m^6^A-circHECA was identified to facilitate the differentiation of SHF stem cells into hair follicle lineage through miR-449a-5p/LEF1 mediated Wnt/β-catenin pathway. Although it is not yet known whether there is other model by which m^6^A-circHECA is implicated in Wnt/β-catenin pathway, our results implied that m^6^A-circHECA positively regulated the Wnt/β-catenin pathway by sponging *miR-449a-5p* mediated *LEF1* upregulation in SHF stem cells of cashmere goats. Thus, we elucidated a novel molecular pathway: m^6^A-circHECA/miR-449a-5p/LEF1/Wnt/β-catenin in the differentiation of SHF stem cells toward hair follicle lineages (Figure 6), and provided evidences for m^6^A-circHECA to act as a potential regulating target to the differentiation of SHF stem cells in cashmere goats. In this work, however, we must point out that, firstly, the current study was conducted in SHF stem cells of cashmere goats cultured *in vitro*; and secondly, we tested the expression abundance of the analyzed genes at mRNA level, but not at protein level. Thus, the revealed significant role of m^6^A-circHECA along with the molecular mechanism above should be further validated in SHF stem cells of cashmere goats *in vivo* with the expression testing of the involved genes at protein level as suggested in previous publication [55].

## CONCLUSION

The circHECA relying on m^6^A modification to contribute the differentiation of SHF stem cells into hair follicle lineage in cashmere goats, and this revealed functional role of circHECA may be achieved through miR-449a-5p/LEF1 mediated Wnt/β-catenin pathway.

## Figures and Tables

**Table 1:** Detail of PCR primers utilized in this study along with the corresponding annealing temperature for PCR amplification

Genes	Reference	Primer pair with sequence (5’–3’)	Primer length (nt)	Annealing temperature (°C)	Amplicon size (bp)
circHECA (Divergent primers)	Shen et al. [30]	F: ACGATGTTCCCTGTCACCTT	20	57	87
		R: TCGTCTTTCTCCAGGTCCAC	20		
*HECA*	XM_018053273.1 in GenBank	F: CGGGGTTGTCGGTTCATAGAG	21	56	117
		R: CGTGGAAAGTGTTCAGCTTGT	21		
*Keratin 6*	Yin et al. [14]	F: CAGTCGCAGCCTCTACAACCT	21	56	159
		R: CAAATGCCACCTCCATAACCA	21		
*Keratin 7*	Yin et al. [14]	F: GAGTTTGTGGTGTTGAAGAA	20	56	194
		R: AAGTCCAGGGAGCGGTTGTT	20		
*Keratin 8*	Yin et al. [14]	F: TCCTTCAGCAGCCGCTCCTA	20	58	160
		R: CTGTAATGCCCCCCAAACCT	20		
*Keratin 16*	Yin et al. [14]	F: CCTTTGTGGCTAGTGGTATG	20	55	188
		R: CAGTTTCAGGGGTTGCTTAT	20		
*Keratin 17*	Yin et al. [14]	F: GGGGAATGGAAACAGAGGAG	20	56	112
		R: GAGGAGAGAAGCCCAAGATG	20		
*LEF1*	Zhu et al. [15]	F: CCACCTCTTGGCTGGTTTTC	20	56	176
		R: TTTGGCTCCTGCTCCTTTCT	20		
*Survivin*	XM_005694094.3 in GenBank	F: TTCAGCCCCAGCACAGACTA	20	55	216
		R: GCCACACACACAAAAAGCCA	20		
*c-Myc*	XM_018058563.1in GenBank	F: CTCACAGCCCGTTGGTCCTA	20	56	200
		R: CCGCCTCTTGTCATTCTCCT	20		
*β-catenin*	XM_018066894.1in GenBank	F: TGGAGCCAGACAGAAAAGCA	20	54	184
		R: GTGAAGGACTGAGAAAACCC	20		
*Cyclin D1*	XM_018043271.1 in GenBank	F: GCCGAGGAGAACAAGCAGAT	20	58	94
		R: TGGAGGGTGGGTTGGAAATG	20		
*GAPDH*	Yin et al. [28]	F: TGAACCACGAGAAGTATAACAACA	24	53	125
		R: GGTCATAAGTCCCTCCACGAT	21		
*miR-449a-5p*	MIMAT0036230 in miRNAsong	F: CGTGGCAGTGTATTGTTAGCTGG	23	60	Not available
*snRNA-U6*	Yin et al. [28]	F: CGCTTCGGCAGCACATATAC	20	55	Not available
		R: AAATATGGAACGCTTCACGA	20		

PCR, polymerase chain reaction.

## Data Availability

Upon reasonable request, the datasets of this study can be available from the corresponding author.
